# Copper price prediction using LSTM recurrent neural network integrated simulated annealing algorithm

**DOI:** 10.1371/journal.pone.0285631

**Published:** 2023-10-30

**Authors:** Jiahao Chen, Jiahui Yi, Kailei Liu, Jinhua Cheng, Yin Feng, Chuandi Fang

**Affiliations:** 1 School of Economics and Management, China University of Geosciences, Wuhan, Hubei, China; 2 Economics & Technology Research Institute, China National Petroleum Corporation, Beijing, China; 3 School of Lowcarben Economics, Hubei University of Economics, Wuhan, Hubei, China; 4 Collaborative Innovation Center for Emissions Trading System Co-Constructed by the Province and Ministry, Wuhan, China; 5 Law and Business School, Wuhan Institute of Technology, Wuhan, Hubei, China; Univerzitet Singidunum, SERBIA

## Abstract

Copper is an important mineral and fluctuations in copper prices can affect the stable functioning of some countries’ economies. Policy makers, futures traders and individual investors are very concerned about copper prices. In a recent paper, we use an artificial intelligence model long short-term memory (LSTM) to predict copper prices. To improve the efficiency of long short-term memory (LSTM) model, we introduced a simulated annealing (SA) algorithm to find the best combination of hyperparameters. The feature engineering problem of the AI model is then solved by correlation analysis. Three economic indicators, West Texas Intermediate Oil Price, Gold Price and Silver Price, which are highly correlated with copper prices, were selected as inputs to be used in the training and forecasting model. Three different copper price time periods, namely 485, 363 and 242 days, were chosen for the model forecasts. The forecast errors are 0.00195, 0.0019 and 0.00097, respectively. Compared with the existing literature, the prediction results of this paper are more accurate and less error. The research in this paper provides a reliable reference for analyzing future copper price changes.

## Introduction

Copper, a critically important metal, has always been a concern of policy-makers. For example, Chile, the largest exporter of copper in the world, produced 28% (estimated 5.6 million metric tons) of the global copper production in 2019 [1]. The copper price is the economic cornerstone for Chile. According to the estimation of copper demand, the demand for copper will significantly increase by between 275% and 350% by 2050 [2]. Some countries’ economies rely on copper. The fact that the copper price increases or decreases has a profound influence on some countries’ economies. In addition, as a kind of financial subject matter, copper futures are widely traded. There are hundreds of millions of institutional and individual investors all over the world. Through copper futures contracts, speculation, arbitrage, and hedge trades are undergoing. Hence, for a long time, financial researchers have been devoted to studying how to efficiently and precisely forecast the price of copper to help them avoid risk and make profit.

Traditionally, the copper price can be forecasted through the statistical time series model. One of the basic models is the autoregressive integrated moving average (ARIMA) model. Although the ARIMA model works easily [3–5], it can only be used to capture the linear relationship with time series. In addition, time series must be stationary, which requires that the mean and variance of the time series not dramatically fluctuate. Although differencing transformation is implemented to ensure stationarity, the times of differencing transformation are unknown. All of these factors pose an obstacle to applying the ARIMA model. Considering the different standard deviations of the yield rate during different periods of time, the generalized autoregressive conditional heteroskedasticity (GARCH) model is introduced to predict the time series variance [6, 7], but the weakness of GARCH is that the model is symmetric in terms of modeling volatility. In the real market, volatility increases in the downward trend of returns with bad news, and volatility decreases in the upward trend of returns with good news [8]. GARCH cannot be used to explain this kind of effect.

As artificial intelligence is developed so fast, advanced algorithms have already begun to be applied in the price forecasting field. Usually, the overarching advantage of AI technology is that it works nonlinearly; in other words, AI can simulate any nonlinear function because the activation function differs from traditional simple perceptrons. A feed-forward neural network (FFNN), for example, is used to forecast uranium prices [9]. A back propagation (BP) neural network is also used to predict stock prices [10, 11]. However, a data point of time series is not isolated; in other words, a historical time point can contribute to the current price, which is the cornerstone of time series analysis. Researchers believe that there is autocorrelation in the stationary time series after trend removal. FFNN and BP cannot deal with this kind of data series very well. A recurrent neural network (RNN) can predict this kind of time series with good result [12–14]. This is because the hidden layer of RNN involves the information from the previous moment, which treats the time series as a whole part rather than a single point. The weakness of RNN is gradient vanishing. The information is passed moment by moment using factorial multiplication. However, after many multiplication operations, the value of the historical information weight will approach 0, which means that the current moment only depends on the nearby moments. When the range of data is years, RNN does not work effectively.

A long short-term memory (LSTM) neural network is introduced to solve the vanishing gradient problem [15]. There is a cell state line crossing the whole time series, and there are three special gates (forget gate, input gate and output gate) in the LSTM. At each single time point, unnecessary information is eliminated from the cell state, and new information is added to the cell state. Through the addition operation rather than the multiplication operation, every single moment of valid information can be conserved. Long-term memory can be withdrawn. Compared with the RNN, LSTM can provide a more accurate prediction of time series. In recent years, LSTM has become popular in the price forecasting field [16–21]. For example, predicting the close price of a stock market [22], combining LSTM and the salp swarm algorithm to improve accuracy of crude oil price forecasting [23], LSTM and multi-head attention to forecast a given time series [24], graph Long Short-Term Memory (GLSTM) neural network to predict the air quality characteristics [25].

In the field of copper price forecasting, many scholars have carried out innovative research. Such as bat algorithm (BA) [26], support vector regression (SVR) [27], adaptive neuro-fuzzy inference system (ANFIS) [28]. However, the convergence and stability of BA need to be further improved. When solving nonlinear problems, SVR is difficult to choose a suitable kernel function. ANFIS is convenient and efficient, while the lack of adaptive capability of fuzzy systems limits its application. Therefore, long short-term memory (LSTM) also drew a lot of attention [29–31].

Although LSTM works well in forecasting, one of the common problems of artificial intelligence models is tuning hyperparameters. Hyperparameters include the batch size, number of neurons, number of hidden layers and so on [32]. Hyperparameters can dramatically influence the performance of the AI model. Traditionally, grid searching and random searching are methods to address this issue. Grid searching is too time-consuming, and random searching could miss the optimal solution. Researchers have attempted to use many other methods, for example, particle swarm optimization [14, 33], beetle antennae search algorithm (BAS) [34], teaching and learning based optimization (TLBO) model is applied to tune the output unit of the LSTM [35]. A common problem in other methods is that the local optimal solution can be found easily, and the global optimal solution is most likely missed. The simulated annealing (SA) algorithm can be used to sufficiently address this problem. In other words, SA allows the acceptance of a bad set of parameters under a probability at the beginning, which means that every direction existing in the optimal solution will be searched. At the end of all iterations, the solution is probably the global best solution.

Another key point that makes an AI model successful is feature engineering. Many factors influence the price of copper. An AI model cannot consider all of these factors, resulting in the “curse of dimensionality”. However, too few features can cause insufficient machine learning. Researchers have attempted to combine principal component analysis (PCA) and AI models [36], but PCA focuses on dimension reduction; furthermore, it is a linear transformation. As long as the number of dimensions is not extremely large, PCA is not necessary. Instead, a few of the most relevant features can be selected. Hence, correlation analysis can work sufficiently well.

In the current paper, our innovation is to use simulated annealing (SA) algorithm to find the best set of hyperparameters for the LSTM model, which makes the LSTM model more efficient and accurate. Meanwhile, correlation analysis is used to solve the feature engineering problem of AI models. Then three economic indicators, WTI crude oil price, closing gold price and closing silver price, are used as the inputs of the model. In the model prediction, three different copper price time periods, 485 days, 363 days and 242 days, are chosen. The prediction error is 0.00195, 0.0019, and 0.00097, respectively.

The subsequent parts of this paper are organized as follows: Part two introduces the research methods, Part three describes the data processing process, Part four is the analysis and discussion of the prediction results, and Part five is the conclusion and future outlook of the study.

## Methodologies

### Long short-term memory (LSTM)

In recent years, artificial intelligence technologies have become increasingly popular. Basically, the recurrent neural network (RNN) is good at dealing with the time series problem because of the special cell structure. In detail, the information held by the previous neural cell state could contribute weight to the current cell state. This special phenomenon allows RNNs to tackle the time series problem, for example, the natural language process.

Compared with the RNN, the neural cell of LSTM can memorize more historical information because the vanishing gradient existing in the RNN is eliminated in LSTM. Technically, weight propagation is completed through addition operations in LSTM rather than factorial multiplication, which means that the weight will not approach 0 and the information can be retained.

**Fig 1:**
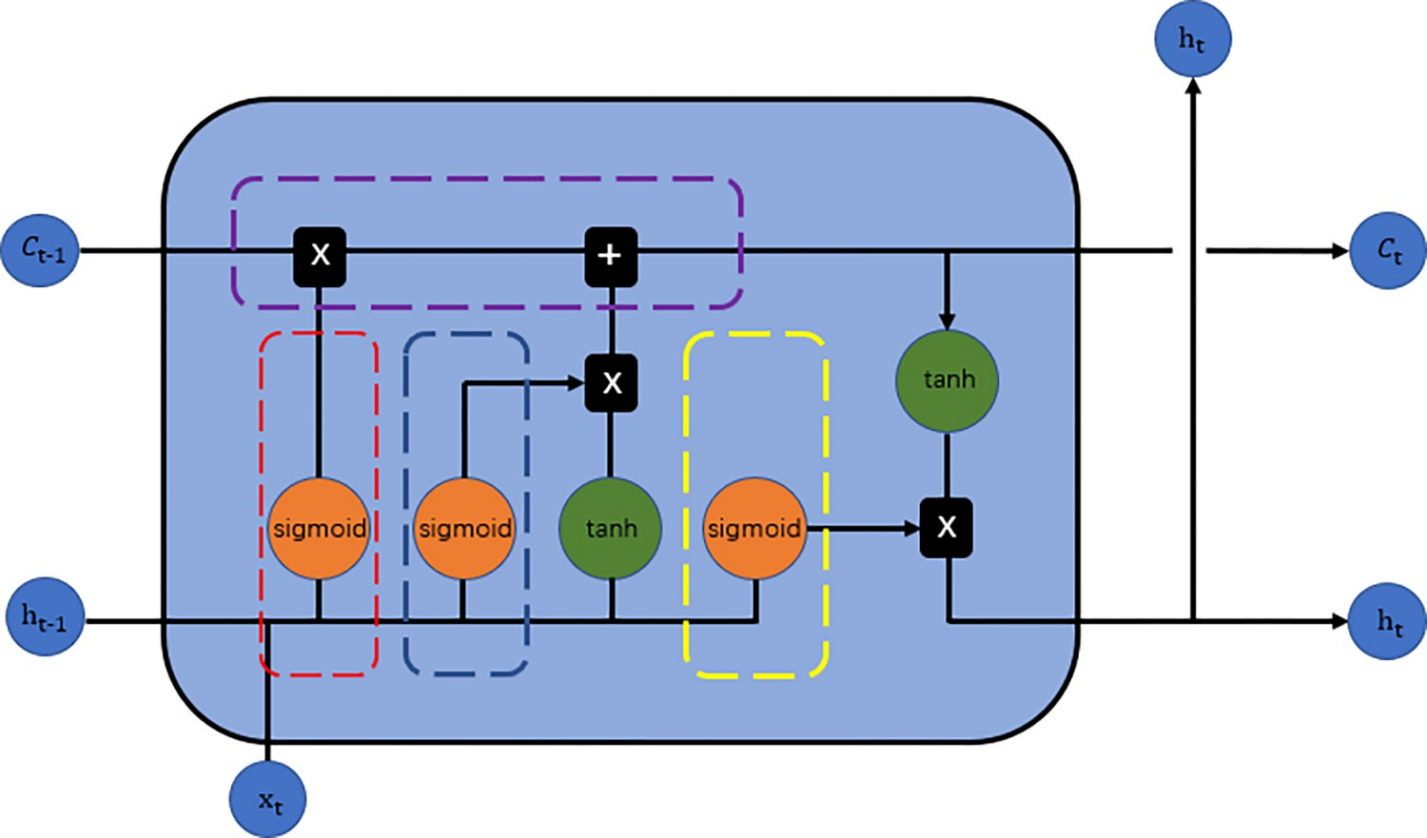
The internal structure of the LSTM cell.

Fig 1 shows the scheme of the LSTM cell. There are several gates in the LSTM that make it special. A gate is a function that gives a result ranging from 0 to 1. A value of 0 means that no information passes through the gate, and a value of 1 means that all information passes through the gate. The gates decide which information should be passed to the next moment and which information is forgotten. However, there are some processes that are used to connect these three gates.

### Forget gate

The forget gate is represented by the red dashed rectangle in Fig 1. First, the output from the last moment, *h*_*t*−1_, concatenates with the new input *x*_*t*_ to a larger vector. Through this gate, the proportion of forgotten information can be obtained. Eq 1 shows this process mathematically.

$$f_{t}=sigmoid(W_{f}*[h_{t-1},x_{t}]+b_{f})$$ (1)

where *f*_*t*_ is the proportion of the forgotten information, sigmoid is the activation function, *W*_*f*_ is the weight matrix forget gate, [*h*_*t*−1_, *x*_*t*_] is the concatenation of vectors *h*_*t*−1_ and *x*_*t*_, and *b*_*f*_ is the bias matrix of the forget gate.

### Input gate

The input gate is the represented by the blue dashed rectangle in Fig 1. The output from the last moment, *h*_*t*−1_, is concatenated with the new input *x*_*t*_ to a larger vector. Through this gate, the proportion of current cell information will be retained. Eq 2 shows this process mathematically.

$$i_{t}=sigmoid(W_{i}*[h_{t-1},x_{t}]+b_{i})$$ (2)

where *i*_*t*_ is the proportion of new information retained, sigmoid is the activation function, *W*_*i*_ is the weight matrix of the input gate, [*h*_*t*−1_, *x*_*t*_] is the concatenation of vectors *h*_*t*−1_ and *x*_*t*_, and *b*_*i*_ is the bias matrix of the input gate.

### Current memory

The dark dashed brown rectangle represents the current memory in Fig 1. This memory includes both unnecessary information and important information. Eq 3 mathematically shows this process.

$${\overset{ˇ}{c}}_{t}=tanh(W_{c}*[h_{t-1},x_{t}]+b_{c})$$ (3)

where ${\overset{ˇ}{c}}_{t}$ is the current memory, tanh is the activation function, *W*_*c*_ is the weight matrix of the current memory, [*h*_*t*−1_, *x*_*t*_] is the concatenation of vectors *h*_*t*−1_ and *x*_*t*_, and *b*_*c*_ is the bias matrix of the current memory.

### Renewing the current cell state

Through the input gate and forget gate, the cell state can be renewed, as shown by the purple box in Fig 1. This cell state will retain the information to the next moment. Eq 4 also shows that information from the input gate is added to the cell state. This addition operation ensures that valid information can be memorized by the model in each moment, which is an important characteristic of the LSTM model.

$$c_{t}=f_{t}*c_{t-1}+i_{t}*{\overset{ˇ}{c}}_{t}$$ (4)

where *c*_*t*_ is the current cell state, *f*_*t*_ is the proportion of forgotten information, *c*_*t*−1_ is the cell state of the last moment, *i*_*t*_ is the proportion of new information retained, ${\overset{ˇ}{c}}_{t}$ is the current memory, and tanh is the activation function.

### Output gate

The output gate is represented by the yellow box in Fig 1. The amount of information that should be output at a given moment is dependent on this gate. Similarly, Eq 5 shows this process mathematically. The result is a proportion value.

$$o_{t}=sigmoid(W_{o}*[h_{t-1},x_{t}]+b_{o})$$ (5)

where *o*_*t*_ is the proportion value that tells the cell state what information should be outputted at this moment, sigmoid is the activation function, *o*_*t*_ is the weight matrix of the output gate, [*h*_*t*−1_, *x*_*t*_] is the concatenation of vectors *h*_*t*−1_ and *x*_*t*_, and *b*_*o*_ is the bias matrix of the input gate.

### Output of the current moment

In the last step, the current cell state is multiplied by the output value from the output gate. The current cell state includes all of the information from the beginning moment to the current moment. Hence, the output of this moment considers all of the given conditions. Eq 6 shows this process mathematically.

$$h_{t}=o_{t}*tanh(c_{t})$$ (6)

where *h*_*t*_ is the output of the current moment, tanh is the activation function, and *c*_*t*_ is the current cell state. Through the above description of the scheme of the internal LSTM neural cell, it is clear that the LSTM model can predict time series considering historical information. Moreover, the behavior of LSTM is much better than that of the RNN.

### Simulated annealing algorithm

Although many artificial intelligence models have been successfully used in the economy field, one of the quintessential problems of these models is hyperparameter searching. In the present paper, the simulated annealing (SA) algorithm is applied to search the best set of hyperparameters. Compared with other optimization algorithms, one of the most atypical advantages of SA is that the global optimal solution can be found easily, which means that the LSTM model can run more efficiently.

**Fig 2:**
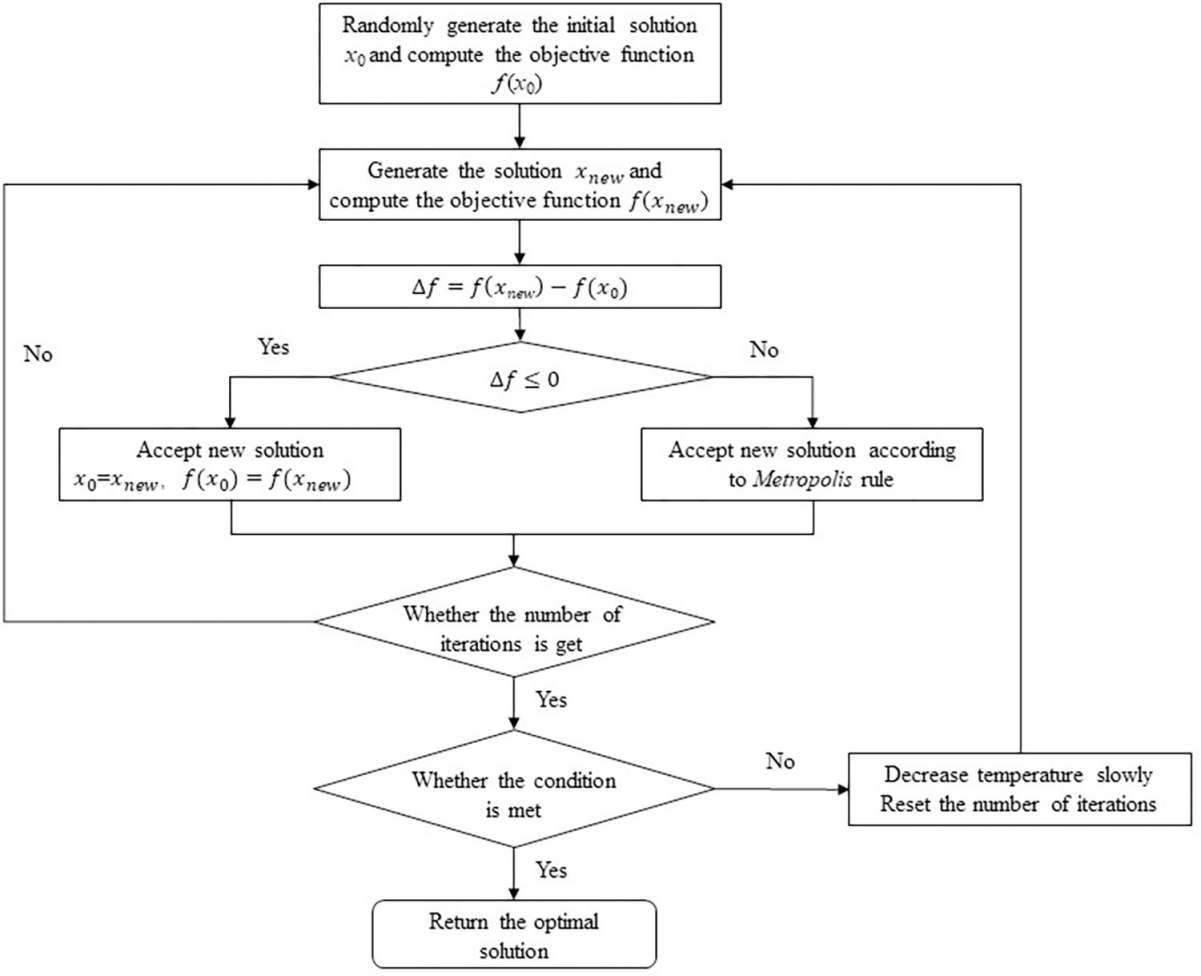
Flow chart of the simulated annealing algorithm [37].

Fig 2 demonstrates the flow of the simulated annealing algorithm, the steps are as follows:

#### Step 1

Set the initial parameters of the artificial intelligence model and SA model. The deadline conditions (final temperature T and iteration number i) of the SA model are also set.

#### Step 2

According to the initial parameters, the new set of parameters can be calculated by using Eq 7.

$$x_{temporary}=x_{old}+T_{current}*u$$ (7)

where *x*_*temporary*_ is the temporary new parameter, *x*_*old*_ is the parameter of the last step, *T* is the current temperature, and *u* is a random number ranging from -1 to 1. Before the first step, *x*_*old*_ and *T*_*current*_ are the initial values.

Because the hyperparameters are in a range, the value of *x*_*temporary*_ must be processed to be in an acceptable range. The sigmoid function is monotonically increasing and ranges from 0 to 1. The sigmoid function is called to obtain the new hyperparameter (Eq 8).

$$x_{new}=a*\frac{1}{1+e^{-x_{temporary}}}$$ (8)

where *x*_*newed*_ is the processed *X*_*temporary*_, and *a* is the artificial setting range.

#### Step 3

Both *x*_*new*_ and *x*_*old*_ are substituted into the model, and then two error values for each set of hyperparameters are calculated by the error function. The difference between these two error values can be represented by the symbol Δ*E* in Eq 9.


$$\Delta E={error}_{from\;new\;x}-{error}_{from\;old\;x}$$ (9)

There are two different situations of Δ*E* (metropolis rule).

- Δ*E*<0
  This situation illustrates that the error calculated from the model result run by *x*_*new*_ is smaller than the error from the last step. In other words, the hyperparameter of the model is improved. Hence, the new set of hyperparameters is kept replacing the old hyperparameters.
- Δ*E*>0
  This situation illustrates that the error calculated from the model result run by *x*_*new*_ is larger than the error from the last step. However, to avoid the local optimal value being accepted, the model needs to be run based on this poor set of hyperparameters. The possibility that the model accepts the poor hyperparameters can be calculated by Eq 10

$$possibility=e^{\left(\frac{\Delta E}{T_{current}}\right)}$$ (10)

where *possibility* is the possibility that a poor set of hyperparameters could be accepted, Δ*E* is the error difference between the new model and the old model, and *T*_*current*_ is the current temperature.

It can be seen that the poor set of hyperparameters can be easily accepted at high temperature, which means that the global optimal parameters are more likely to be found.

#### Step 4

The temperature will be renewed by Eq 11. Step 1 will be performed again until the deadline condition is reached.

$$T_{current}=T_{current}*D$$ (11)

where D is the cool down coefficient and is set to 0.97.

## Data preprocessing

### Data acquisition

For the prediction of the copper price, four groups (eight different economic indicators) are chosen and shown in Table 1. Through the statistical analysis, the high correlation indicators with copper price are chosen and used in the LSTM forecasting model.

**Table 1:** Selected economic indicators.

Group	Economic Indicator
Stock Market Trends	Dow Jones Index
	Nasdaq Index
Dollar Influence	Dollar Index
	10 years T-bond yield rates
Other Metals and Industry Product	Gold Price
	Silver Price
	WTI Crude Oil Price
Trade Volume of Copper	Trade Volume of Copper

All data are collected from the Investing website (https://cn.investing.com/commodities/copper-historical-data), and closed price/yield rates are chosen. The period chosen is from January 1^st^, 1990, to December 31^st^, 2009. If one of these eight indicators’ data is missing in the single trade day, all of the data in that day will be deleted. In total, 4870 pieces of data are collected and used in the data analysis.

Table 2 shows descriptive statistics of the features. The mean closed copper price is 1.38 dollars with a standard deviation of 0.87.

**Table 2:** Loss graph of the LSTM model from different steps of the simulated annealing model.

Feature Name	Mean	Std.Dev	Min	Max
closed_copper_price	1.38	0.87	0.60	4.08
volume_copper	606.82	992.52	0	13130.00
close_dollar_index	92.68	10.50	71.30	121.21
close_t_bill	5.56	1.42	2.06	9.07
close_Nasdaq	1651.42	873.36	325.44	5048.62
close_Dow_Jones_Index	7892.99	3325.25	2365.10	14164.53
close_wti_oil	35.03	24.15	10.72	145.29
close_gold	428.56	180.40	253.00	1018.90
close_silver	6.66	3.52	3.51	20.69

### Feature selection

**Fig 3:**
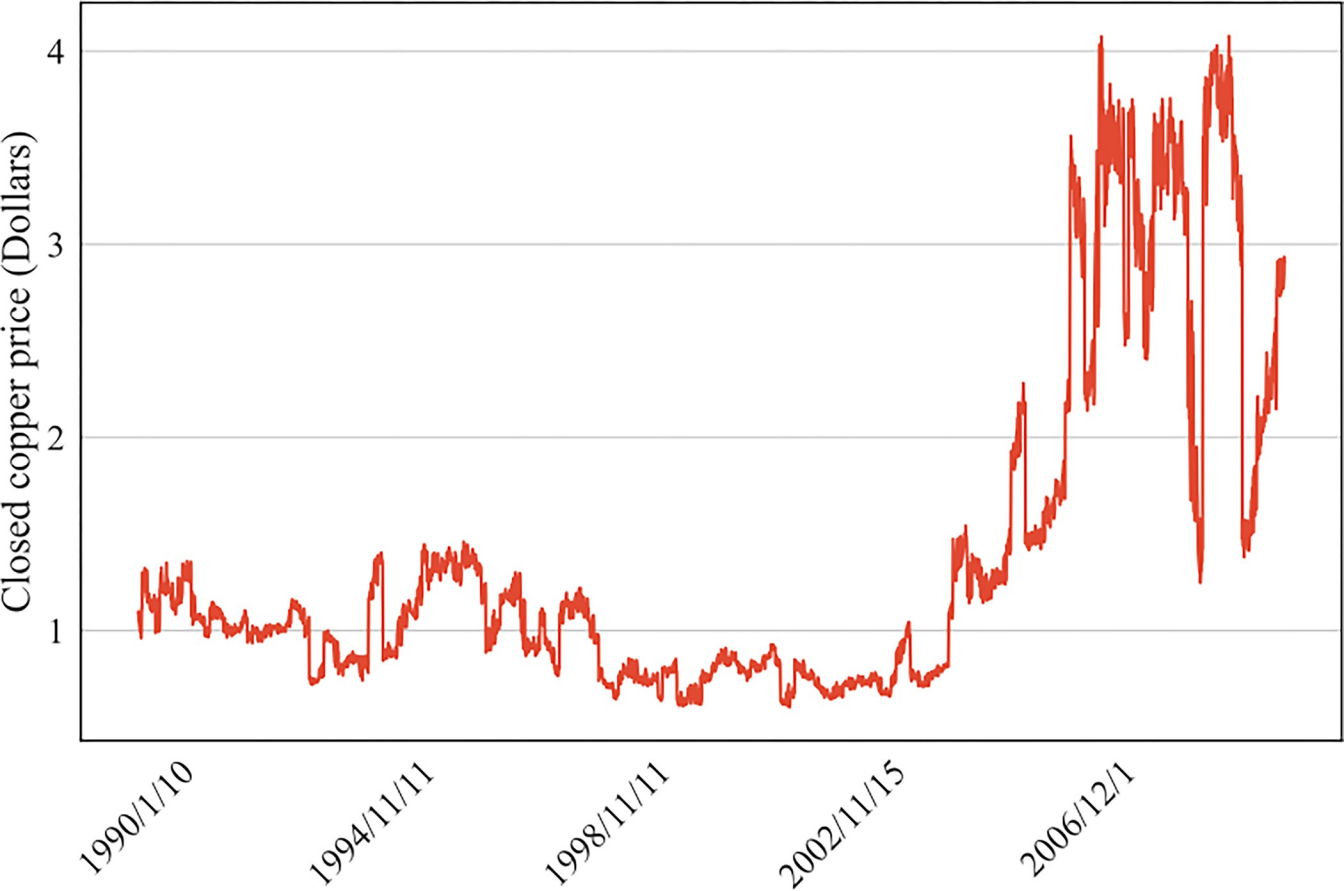
Copper closing price.

**Fig 4:**
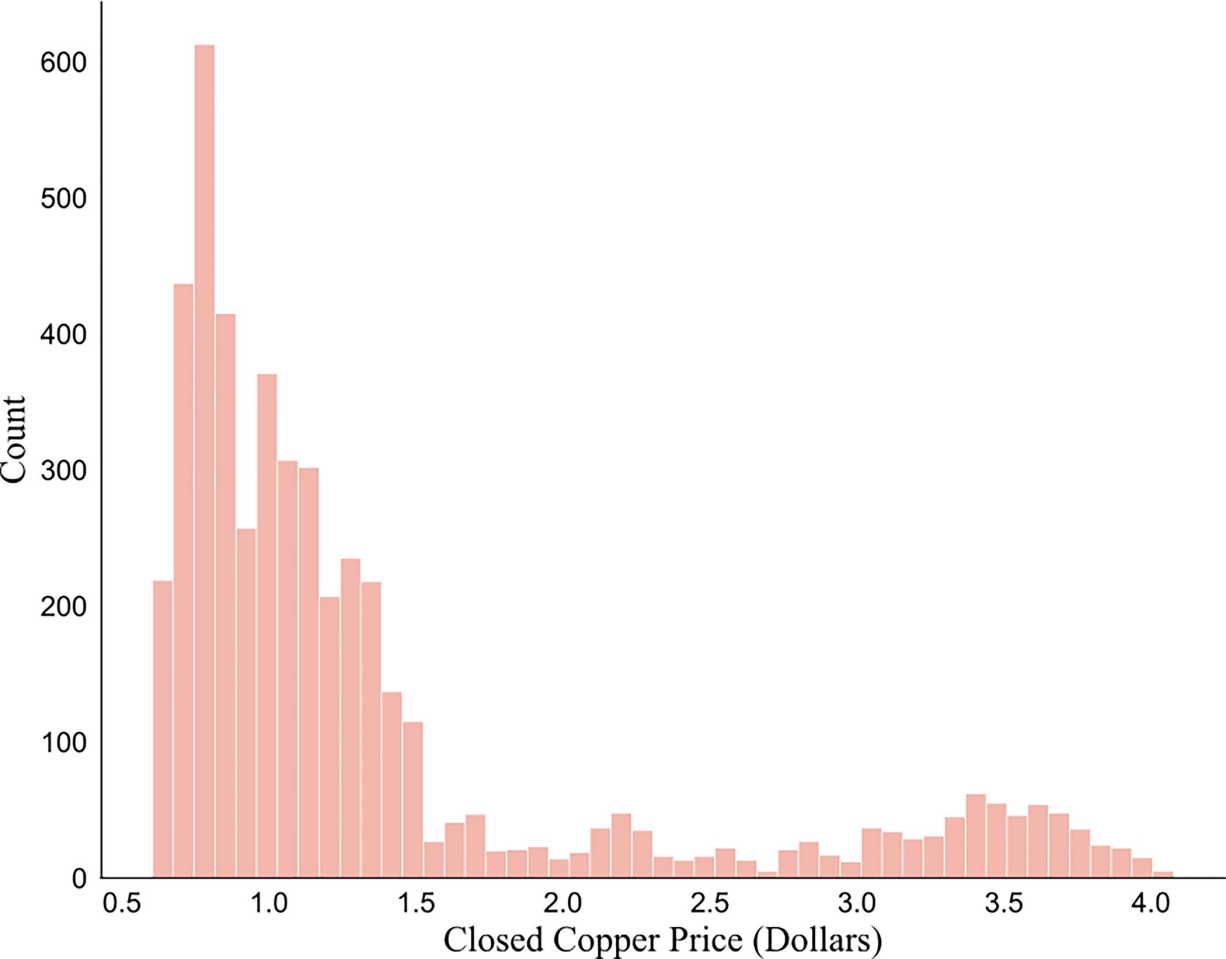
Distribution of copper price.

Fig 3 shows the historical copper price. The mean copper price is $1.38, and the standard deviation is $0.866. Through the KS test, the statistic value is 0.2367, and the p value is 1.6e-237. Hence, it can be seen that the distribution of the copper price (Fig 4) is not a normal distribution, which means that Pearson correlation cannot be used to find the correlation coefficient between the copper price and other economic indicators.

**Fig 5:**
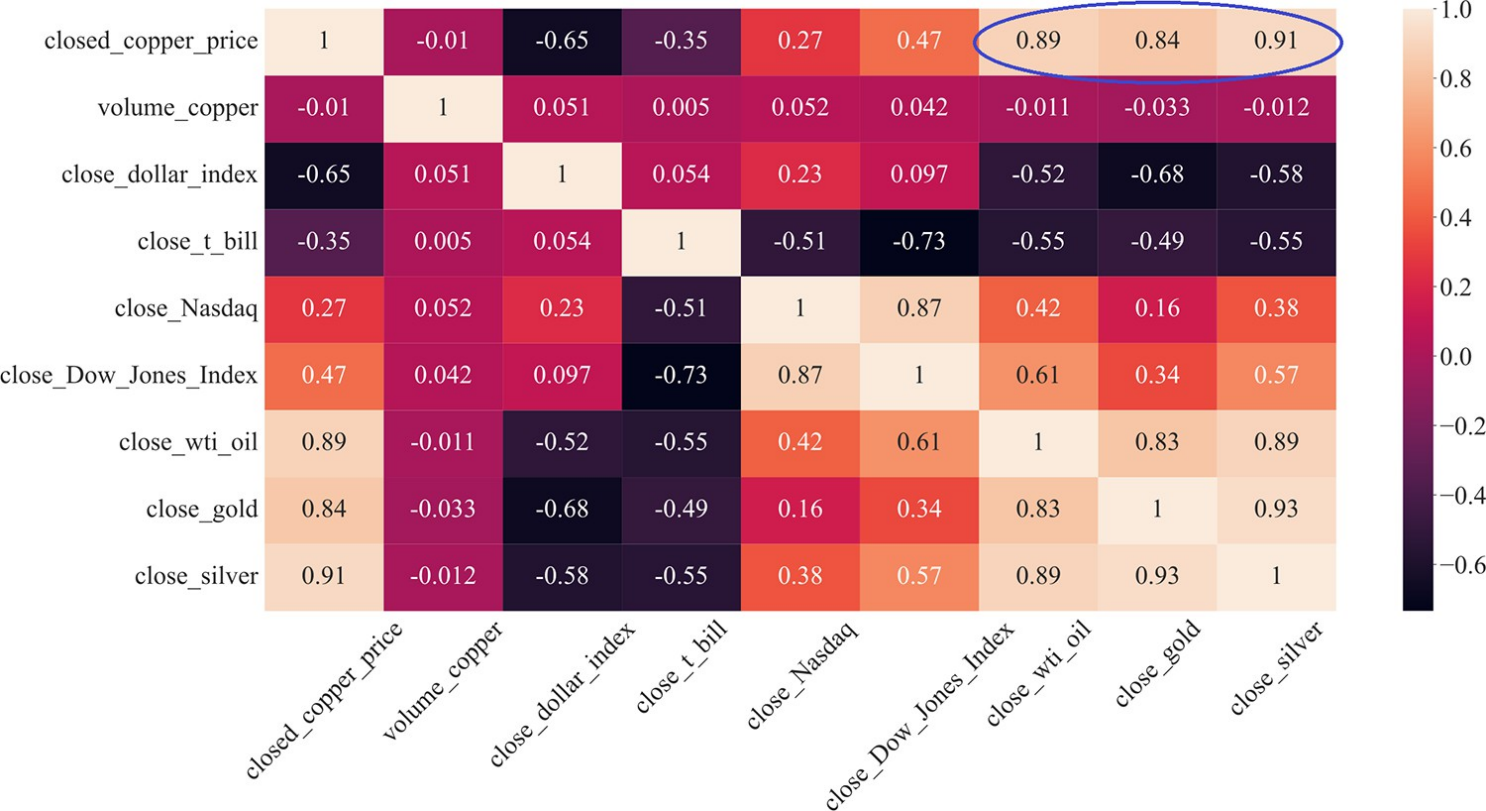
Heatmap of economic indicators.

Because of the nonnormal distribution of copper prices, the correlation coefficient is calculated through the Spearman method. As Fig 5 shows, the Spearman correlation coefficients between the copper price and the WTI crude oil price, closed gold price, and closed silver price are 0.89, 0.84, and 0.91, respectively, which are higher than 0.8. These three economic indices have a strong positive correlation with the copper price. Hence, these three economic indices are chosen as the input of the model.

### Data normalization

To increase the speed of the model running and the model’s accuracy, the chosen data are normalized. All numbers are narrowed down to between 0 and 1 using Eq 12

$${normalized\;x}_{i}=\frac{x_{i}-x_{min}}{x_{max}-x_{min}}$$ (12)

**Fig 6:**
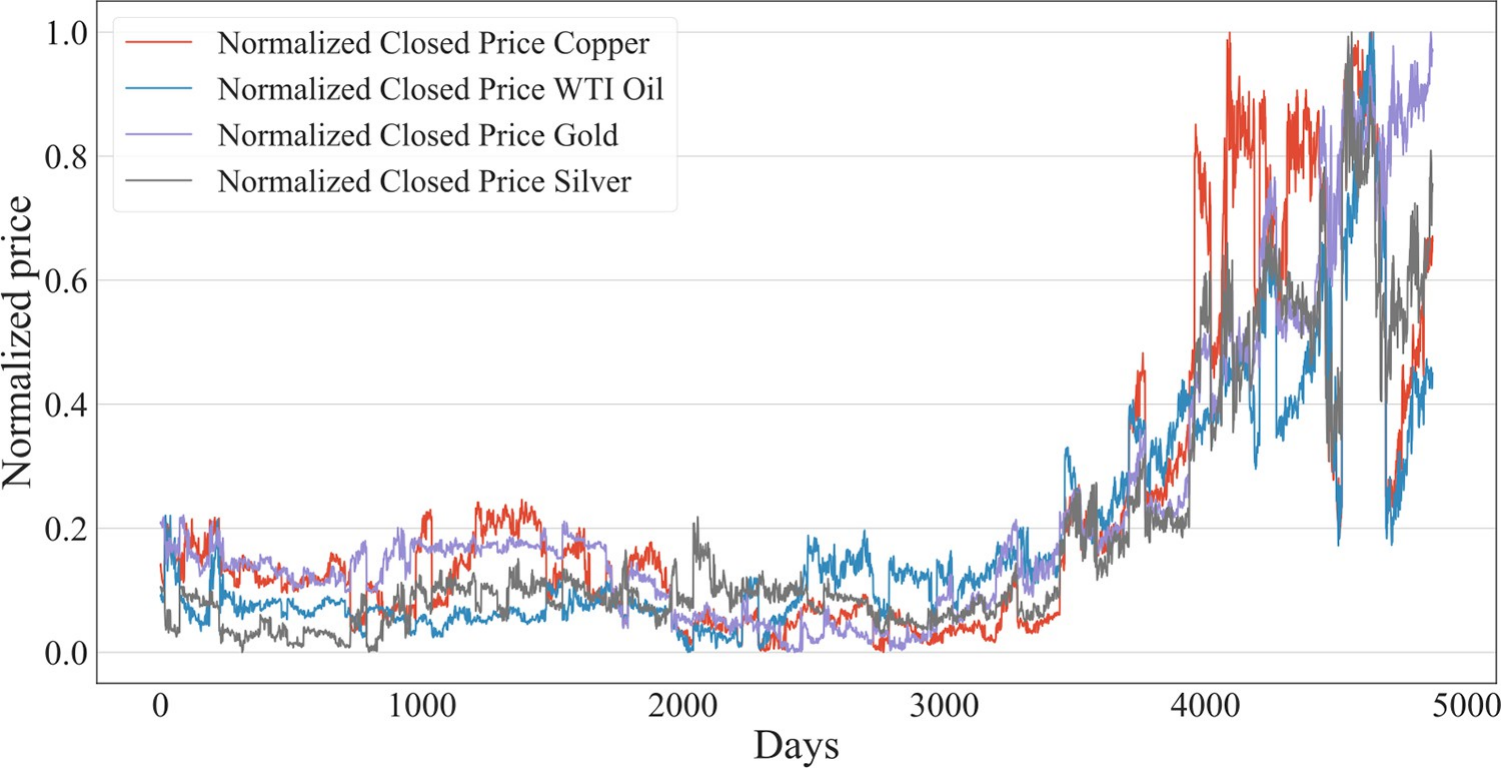
Normalized price trend of chosen economic data.

As Fig 6 shows, these four sets of normalized data have similar trends. These data are split into a training set, validation set and test set.

**Fig 7:**
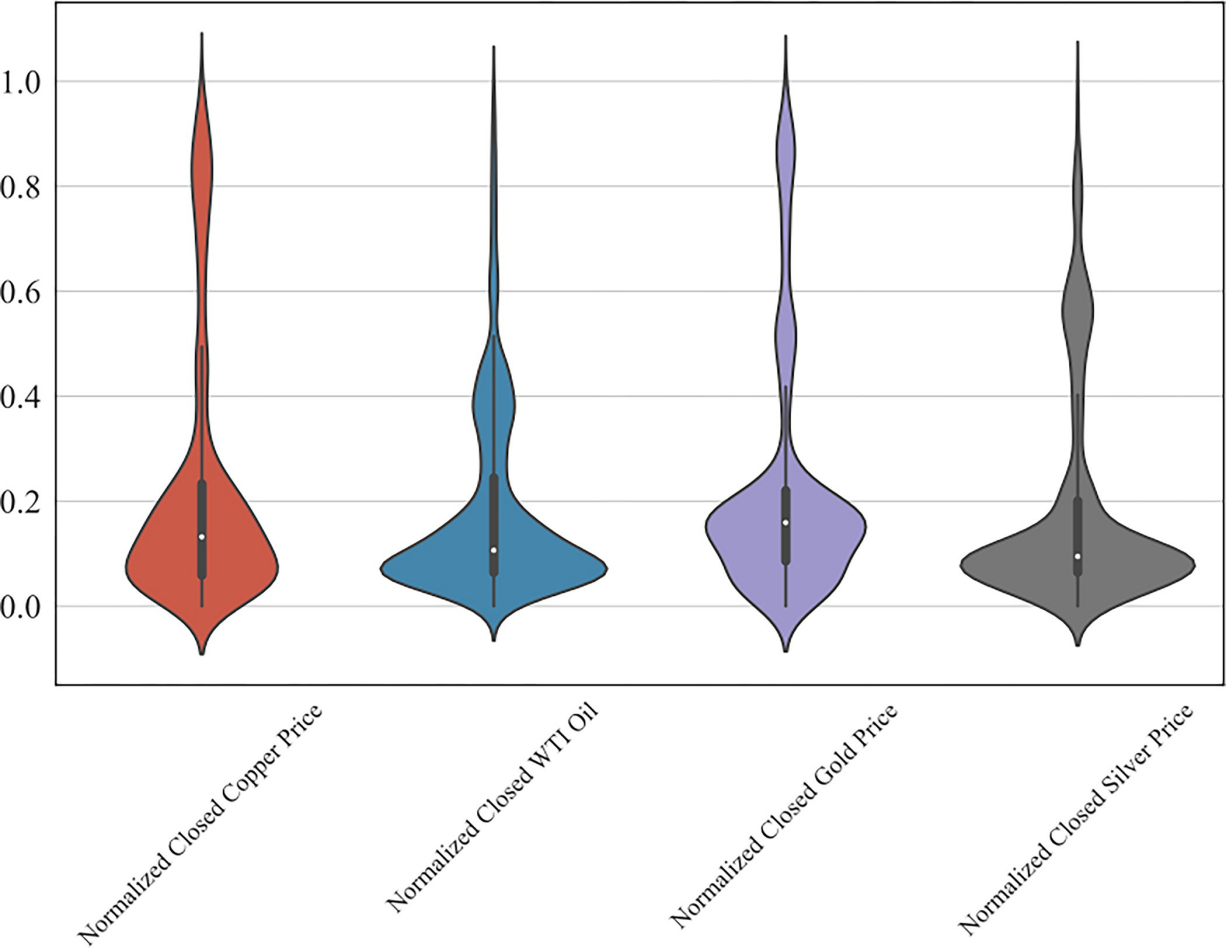
Violin plot after normalization.

As Fig 7 shows, there are only few data points that outside of the 95% confidence interval; however, it is believed that these points represent sudden changes in price and are retained in the dataset.

### Metrics for models

The model will be evaluated by the following formula:

$$MSE=\frac{1}{N_{test}}\sum_{i=1}^{N_{test}}{\left(y_{pred}^{i}-y_{true}^{i}\right)}^{2}$$ (13)

where $y_{true}^{i}$ is the i true value, and $y_{pred}^{i}$ is the i prediction value, *y*_*average*_ is the average of the true value, *N*_*test*_ is the total number of the test data set.

## Discussion

### Simulated annealing algorithm performance

There are hundreds of hyperparameter scenarios for the LSTM model. Finding the most efficient method is challenging. In the current paper, the simulated annealing algorithm is used to perform this task. Before the hyperparameter searching task, the basic and fundamental parameters, which are not changed in the course of the whole prediction process, of the LSTM model are mentioned below. The batch size of the model is 64 and the number of epochs is 100. A dropout layer is introduced into the model in the case of overfitting. The dropout layer is located after the first layer of the dense layer, and the dropout rate is set as 0.2.

**Fig 8:**
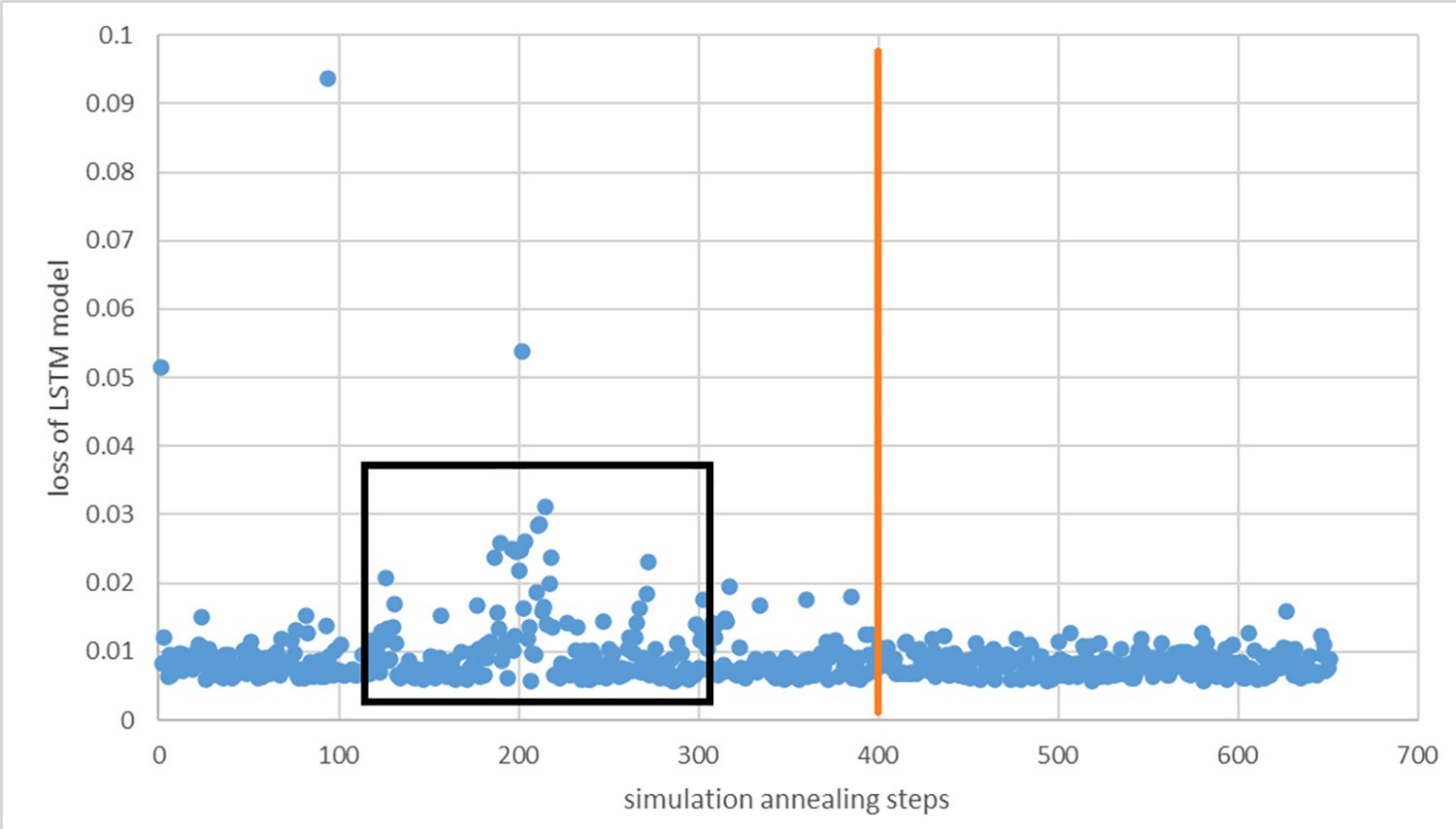
The loss of the LSTM model based on the simulated annealing process.

First, the proposed model is given through the simulated annealing algorithm. The initial temperature, final temperature, and rate of cooling are 1, 0.1, and 0.95, respectively. In addition to these parameters, each simulation process is implemented using 50 iterations. Initially, the learning rate, number of neurons in the first LSTM layer, and number of neurons in the second LSTM layer are set to 0.0005, 15, and 30, respectively. Specifically, the range of the learning rate is set between 0 and 0.005 to ensure that the simulated annealing algorithm can converge. If the learning rate exceeds these bounds, it is set to the default value, 0.00005. The number of neurons must be an integer. In total, there are 650 scenarios in this whole hyperparameter searching job, as shown in Fig 8.

It is clear that the losses of the LSTM model are more discrete before the 400^th^ (before the beige line) simulated annealing step, especially between 120 and 300 steps (black rectangle box). After 400 steps, the loss values of LSTM become stable and plateau at approximately 0.01. The above processing can be seen as a typical simulated annealing process. In other words, the searching algorithm was testing some ‘poor’ scenarios in case the global optimum solution was missed. When cooling down, the model becomes more conservative, the risky options are rejected, and the whole model begins to converge.

**Fig 9:**
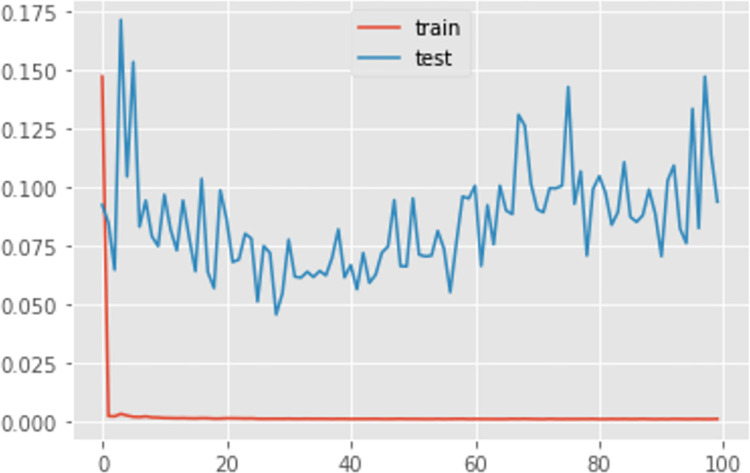
The loss graph of LSTM at 98th steps of the simulated annealing model.

**Fig 10:**
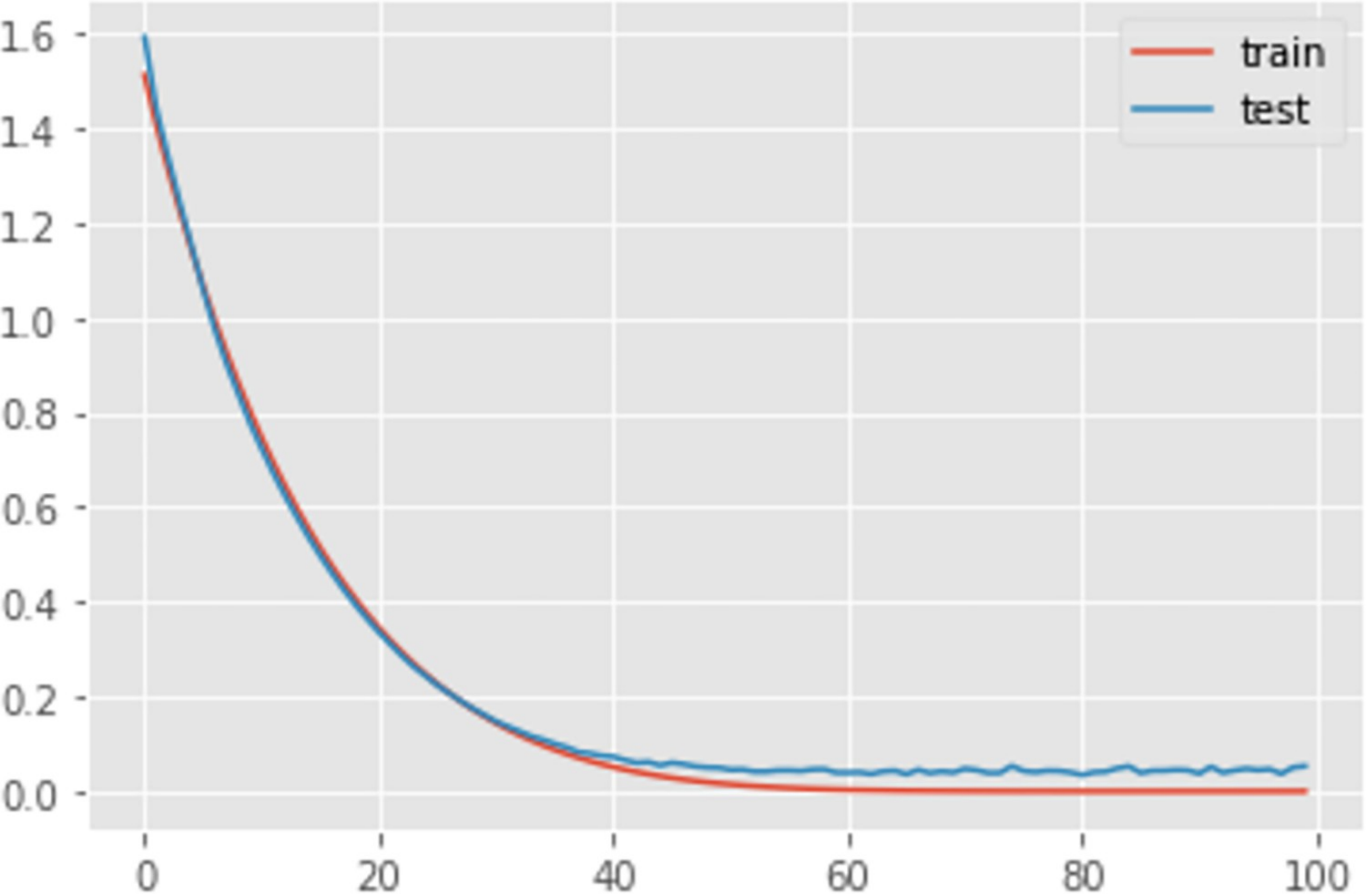
The loss graph of LSTM at 202th steps of the simulated annealing model.

**Fig 11:**
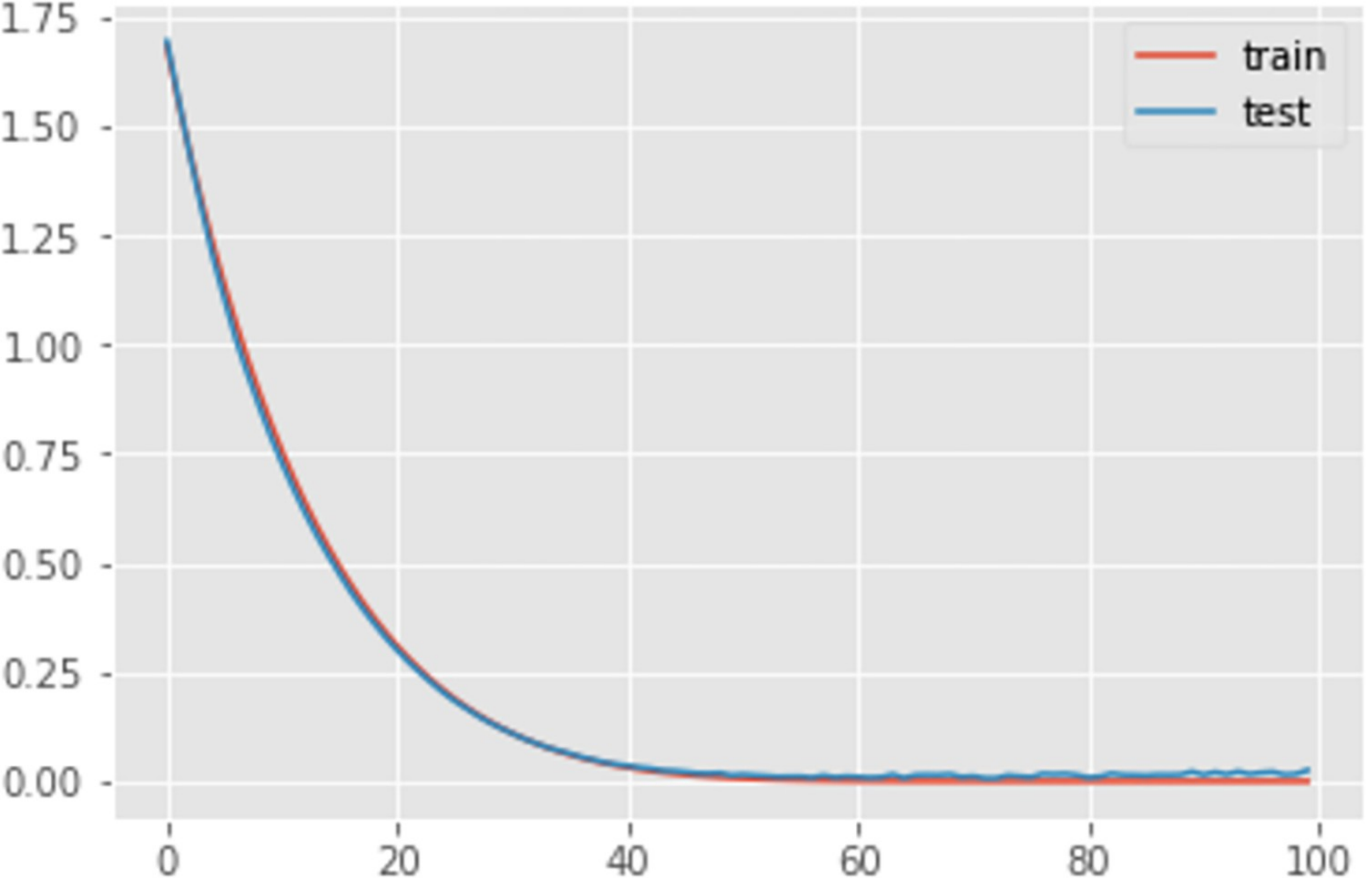
The loss graph of LSTM at 222th steps of the simulated annealing model.

Additionally, Table 3 and Figs 9–11 shows the comparison of the different loss graphs of LSTM at different steps of the simulated annealing model. It can be seen that the worst scenario occurs at 98 steps, for which LSTM loss is 9.37%. Compared with other scenarios, an incorrect numbers of neurons causes this problem. Fortunately, SA finds more and better solutions. For example, at 202 steps, even though the loss is 5.38%, the loss graph looks smoother and more sensible. There is still a small amount of underfitting because the loss from the test is smaller than that from the training set. Until 222 steps, the graph looks good, and the machine learning process is close to perfect.

**Table 3:** Loss graph of the LSTM model at different steps of the simulated annealing model.

Loss Value	SA steps	Scenario	Loss Graph
9.37%	98	Learning rate: 5*10^−5^	Fig 9
		Number of neurons in the first LSTM layer: 73	
		Number of neurons in the second LSTM layer: 82	
5.38%	202	Learning rate: 5*10^−5^	Fig 10
		Number of neurons in the first LSTM layer: 121	
		Number of neurons in the second LSTM layer: 59	
2.61%	222	Learning rate: 5*10^−5^	Fig 11
		Number of neurons in the first LSTM layer: 115	
		Number of neurons in the second LSTM layer: 77	

In the whole hyperparameter searching process, it must be noted that the initial learning rate does not change during the whole process. In other words, the learning rate is always set to the default value (0.00005). An attempt was made to eliminate the bound at the very beginning of the code, but SA crashed, and the loss value of LSTM increased to an unacceptable amount. In fact, many learning rates from outside of the set range were attempted to be used by SA, which is why the learning rate was set to the default value.

### LSTM prediction

At the end of the hyperparameter searching task, the simulated annealing algorithm provides the best scenario. The learning rate is 0.00005, the number of neurons in the first LSTM layer is 39, and the number of neurons in the second LSTM layer is 111. According to this scenario, the loss of the model is reduced to 0.000569. In addition, based on the loss graph, the training of the model is appropriate. There is no underfitting or overfitting. Hence, this scenario of the LSTM hyperparameters can be applied in real prediction work.

**Fig 12:**
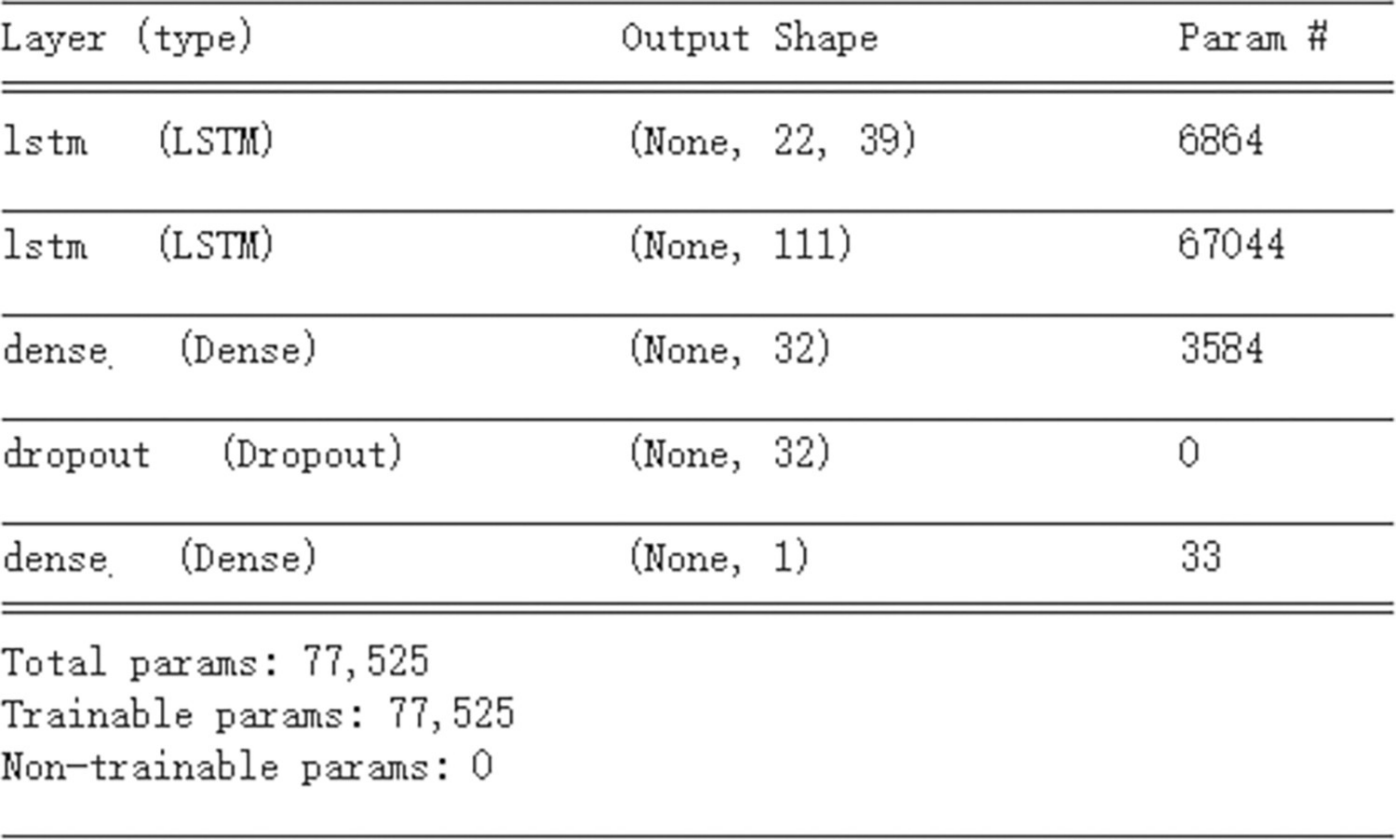
The structure of the LSTM model.

As discussed above, the basic structure parameters of the LSTM model are shown in Table 4 and Fig 12 below.

**Table 4:** Summary of the LSTM model parameters.

LSTM model parameters	
Learning rate	5e-5
Batch size	64
Epochs	100
Dropout rate	0.2
LSTM neurons (first layer)	39
LSTM neurons (second layer)	111

In the prediction process, there are three different time periods of predicted copper prices. In other words, the training data are split by ratios of 0.9, 0.925, and 0.95. The period of the copper price prediction is 485 days, 363 days and 242 days, respectively.

**Fig 13:**
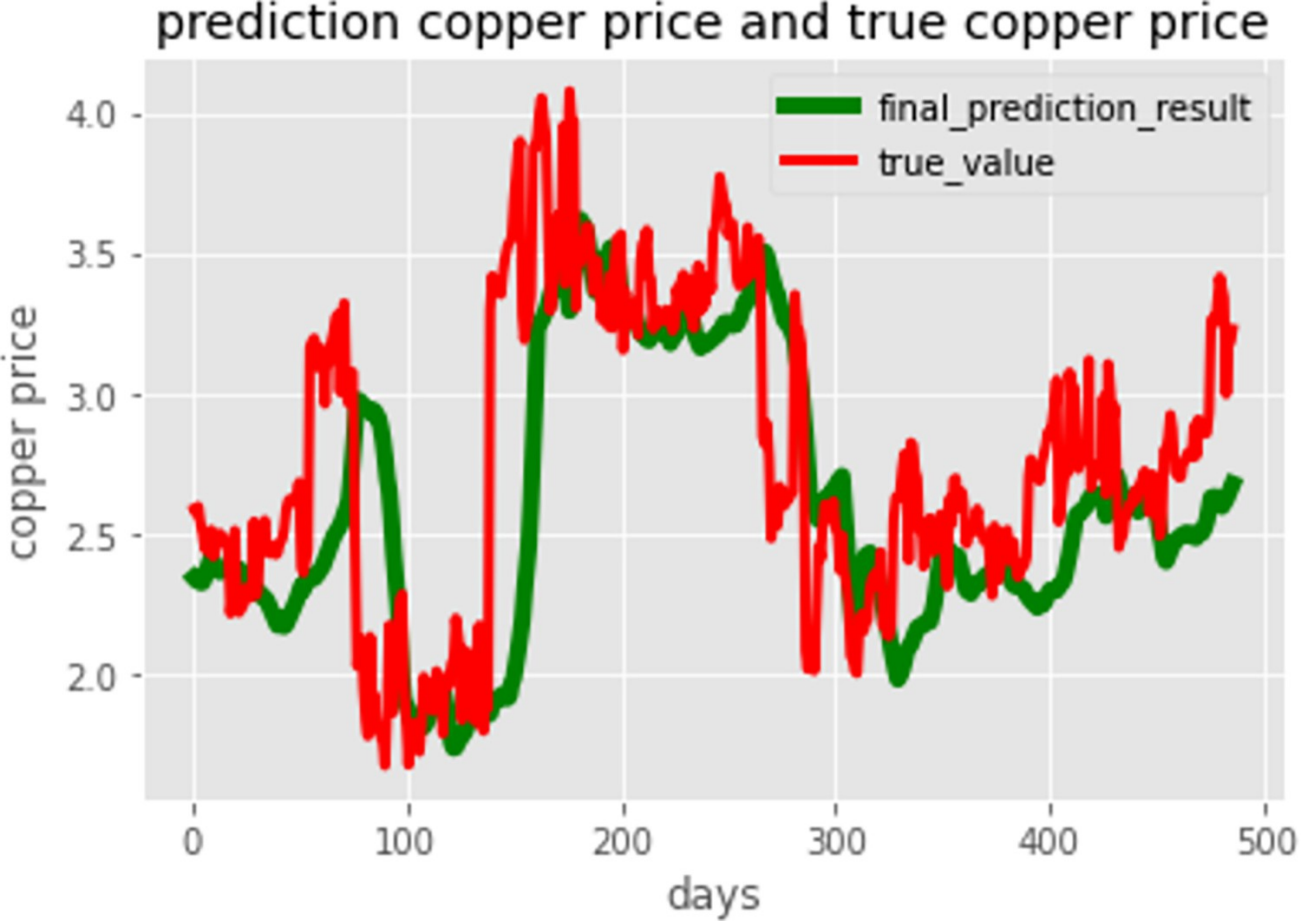
Prediction of the 485-day copper price.

Fig 13 shows the prediction result of the 485-day copper price. Based on this graph, two observations can be made. First, the real price fluctuates more than the predicted price. This could be normal because of noise in the real price data. If a denoising technique is used on these original data, the prediction result could be smoother. Second, the prediction model can provide a relatively good prediction of the copper price. Although a precise price could not be captured by the model, the total trend of the copper price is well represented. The mean square error of the prediction result is 1.95%.

**Fig 14:**
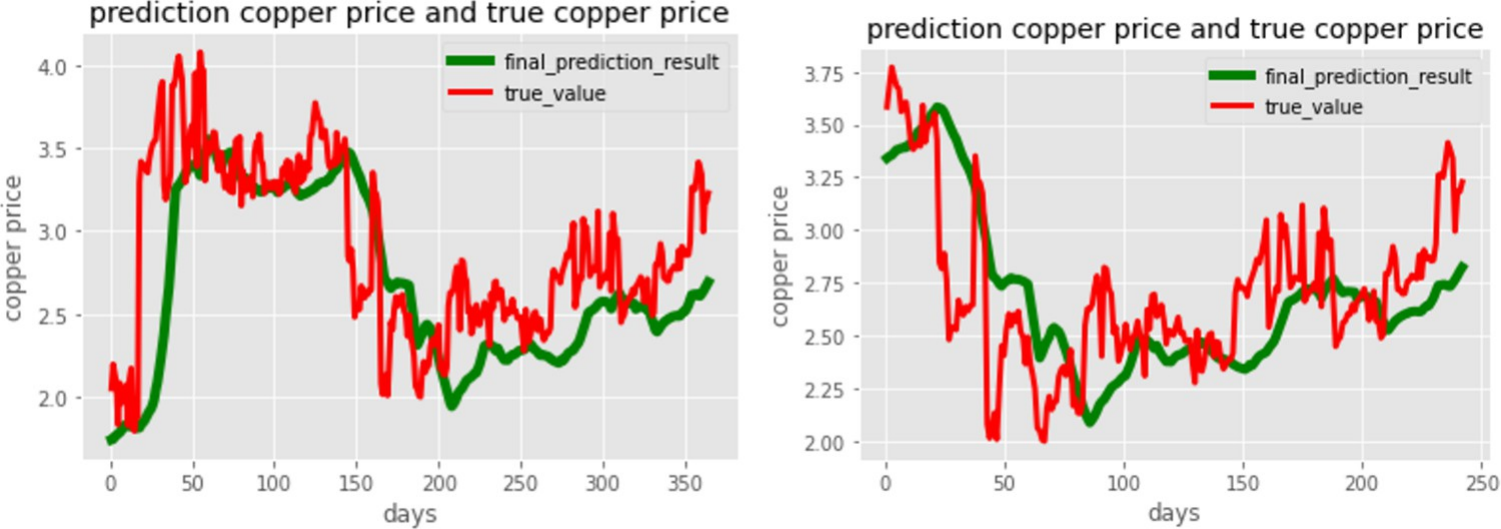
Prediction result of 363-day (left) and 242-day copper price (right).

Fig 14 shows that the copper price trend can be predicted accurately for a shorter time period. The mean square error for these two predictions is 0.0019 and 0.00097. As the prediction time period becomes shorter, the prediction accuracy increases. This is reasonable because there are more data remaining to help the model learn. In other words, more information can be learned by the model when less data enter the test set. However, there is a critical number for this process, and the model is used to predict the 50-day copper price. The error increases again, which means that the model is probably overfitting the data. The dropout rate of the dense layer or any other technique used for overfitting should be applied.

## Conclusion

Copper, a critically important metal in the modern world, is widely used in industry. As more researchers learn about copper, it is important to pay attention to its financial features. One of the attractive points of copper is the copper price. Unlike past prediction methods, there are now more state-of-the-art techniques used in price prediction. One of these techniques is artificial intelligence. As artificial intelligence has become more common to use, researchers have focused on how to make it work sufficiently.

In a recent paper, three tasks were completed. First, through correlation methods, it was found that the WTI oil price, gold price and silver price have a great influence on the copper price. Hence, these three kinds of data were collected to predict the copper price.

The simulated annealing model is used to find hyperparameters of LSTM. The learning rate of LSTM is 5e-5, the number of neurons in the first LSTM layer is 39, and the number of neurons in the second LSTM layer is 111. In the training phase, the data are split by a ratio of 0.9. The proposed model shows the best behavior, and the error is 0.00569.

In the model prediction, three different copper price time periods, 485 days, 363 days and 242 days, are chosen. The prediction error is 0.00195, 0.0019, and 0.00097, respectively. The more data that are used to train the model, the lower the error. In a recent paper, the model yielded a prediction with an error below 2%.

Although the SA-LSTM model has improved the prediction effect of the LSTM model on copper prices, we believe that the model can be further improved. First, due to the presence of noise in the time series, these noises may have a negative impact on price forecasting. We plan to use signal processing techniques, such as wavelet noise reduction, empirical mode decomposition and other methods, to denoise the time series in the future to highlight the characteristics of price trends. In addition, in view of the problem that the LSTM model does not capture the periodicity and seasonal features of the time series model, we plan to combine the LSTM model with the SARIMA model to try to highlight the seasonality of the time series and facilitate the learning of the LSTM model.
